# Comparison of medical costs generated by IBS patients in primary and secondary care in the Netherlands

**DOI:** 10.1186/s12876-015-0398-8

**Published:** 2015-11-26

**Authors:** Carla E. Flik, Wijnand Laan, André J. P. M. Smout, Bas L. A. M. Weusten, Niek J. de Wit

**Affiliations:** 1 Julius Centre for Health Sciences and Primary Care, University Medical Centre Utrecht, Universiteitsweg 100, 3584 CG Utrecht, The Netherlands; 2 Department of Psychiatry and Psychology, St. Antonius Hospital, Soestwetering 1, 3543 AZ Utrecht, The Netherlands; 3 Department of Gastroenterology and Hepatology, Academic Medical Centre Amsterdam, Meibergdreef 9, 1105 AZ Amsterdam, The Netherlands; 4 Department of Gastroenterology, St. Antonius Hospital, Soestwetering 1, 3543 AZ Utrecht, The Netherlands

**Keywords:** Irritable Bowel Syndrome, Chronic Disease, Gastroenterology / GERD / Dyspepsia, Health economics, Primary Care, Medically Unexplained Symptoms

## Abstract

**Background:**

Irritable Bowel Syndrome (IBS) is a functional somatic syndrome characterized by patterns of persistent bodily complaints for which a thorough diagnostic workup does not reveal adequate explanatory structural pathology. Detailed insight into disease-specific health-care costs is critical because it co-determines the societal impact of the disease, enables the assessment of cost-effectiveness of existing and new treatments, and facilitates choices in treatment policy. In the present study the aim was, to compare the costs and magnitude of healthcare consumption for patients diagnosed with Irritable Bowel Syndrome (IBS) in primary and secondary care, compare these costs with the average health care expenditure for patients without IBS and describe these costs in further detail.

**Methods:**

Reimbursement data for patients diagnosed with IBS by a general practitioner (GP) or specialist between 2006 and 2009 were extracted from a healthcare insurance company and compared to an age and gender matched control group of patients without IBS. Using a case-control design, direct medical costs for GP consultations, specialist care and medication prescriptions were calculated.

**Results:**

Data of 326 primary care and 9274 secondary care IBS patients were included in the analysis. For primary care patients, the mean total annual health care costs for the three years after diagnosis compared to the three years before diagnosis, increased with 486 Euro after IBS was diagnosed, whereas for secondary care patients, these costs increased with 2328 Euro. Total health care costs remained higher in the three years after the initial diagnosis when the patient is treated in secondary care, compared to primary care. This increase was significant for hospital specialist costs and medications, but not for GP contacts. For controls, there was no significant difference in mean total annual health costs in the three years before and the three years after the diagnosis and also no significant difference in cost increases between both primary- and secondary-care control patients.

**Conclusion:**

Total healthcare costs per patient substantially increase after a diagnosis of IBS and IBS related costs are significantly higher when patients are treated in secondary-care compared to primary-care. IBS patients should be treated in primary-care where possible, not only because guidelines recommend this from a quality of care viewpoint, but also to optimize use of health care resources. Referral should be restricted to those patients with alarm symptoms, with ill-matching symptoms, or other cases of diagnostic uncertainty.

## Background

Irritable bowel syndrome (IBS) is a functional somatic syndrome (FSS) characterised by patterns of persistent bodily complaints for which a thorough diagnostic workup does not reveal adequate explanatory structural pathology [1]. IBS is one of the most prevalent gastrointestinal disorders in Western countries [2] and is reported to frequently co-occur with other chronic and functional diseases [3]. Similar to individuals with other FSS, IBS patients who seek consultations utilise health care services more frequently than non-IBS patients [4]. IBS causes a substantial economic burden. In two systematic reviews, total direct costs for IBS in the United States and the United Kingdom are estimated between 348 USD and 8750 USD per patient per year [5] with total annual costs of 45.6 million pounds in the UK and 1.35 billion USD in the USA [6]. Detailed insight into disease-specific health care costs is critical because it co-determines the societal impact of the disease, enables the assessment of the cost-effectiveness of existing and new treatments, and facilitates choices in treatment policy [7].

Societal costs for health care can be divided into several categories: direct medical costs associated with diagnosis and treatment, indirect costs related to production losses, and intangible costs related to the impact of the disease on the patient’s quality of life [8].

Although guidelines suggest that in the absence of alarm symptoms, the majority of IBS patients can be adequately managed in primary care [9], there are substantial differences in referral rates across Europe, ranging from 10 % in the Netherlands [10] to 44 % in the UK [5]. If disease-related medical costs for IBS management substantially differ between primary and secondary care, variation in referral rates will have important economic consequences.

In the present study, we aimed to obtain insight into the economic consequences of referral in IBS management by comparing direct medical costs for IBS when patients are diagnosed and treated in primary or secondary care. We hypothesised that IBS management in secondary care is more costly than that in primary care because of increased co-morbidity, more intensive use of diagnostic tests and frequent cross-referral to other specialists.

## Methods

### Design

We performed a retrospective case-control study using reimbursement anonymised data from Achmea Health Insurance, one of the largest health insurance companies in the Netherlands, and anonymised routine care data from the primary care database of the Julius Primary Care Network (JPCN) of the University Medical Centre, Utrecht. In the Netherlands all patients have an compulsory healthcare insurance. The use of the data was approved by the research committees of both Achmea and JPCN. Patients from the JPCN database were linked to an Achmea policy holder by means of chance linking based on date of birth, gender and postal-code. As the data were delivered to the researchers anonymously, no informed consent from the insurees was necessary.

### Databases

The Achmea database contains reimbursement data for more than one million patients. Achmea facilitates the use of anonymised reimbursed healthcare consumption data for scientific research under strict scientific and ethical conditions. These data include medications, GP and specialist consultations, diagnostic and therapeutic procedures and hospital admissions. The reimbursement for diagnostic and therapeutic procedures is standardised using Diagnostic Treatment Codes (Dutch : DBC), which include the first and last dates of treatment, the treating specialist, the type of treatment and the diagnosis. Medications are registered in the database using anatomical therapeutic codes (ATC) detailing the daily defined dosages, the date and the costs of the medication reimbursements. The GP contacts are registered using the date, type of contact, International Classification for Primary Care (ICPC) -coded diagnosis and reimbursed costs.

The JPCN database contains anonymous routine healthcare data extracted from the electronic medical records (EMR) of 140 general practices with approximately 240.000 patients. The JPCN population represents an average Dutch urban population. Approximately 50 % of JPCN patients are insured by Achmea. In addition to the demographic information, the database contains the ICPC base diagnoses, diagnostic results, and ATC-based prescription data.

### Patient selection

We selected patients from 23 JPCN practices that accurately registered the insurance companies used by their patients. Four groups were selected. The first group was drawn from these 23 practices and consisted of all patients who were diagnosed with IBS (ICPC-code) between 2006 and 2009 who were not referred to secondary care and who were insured with Achmea healthcare. This group will be referred to as primary care patients.

The second group consisted of all patients from the Achmea database who were diagnosed with IBS by hospital specialists (DBC-codes) between 2006 and 2009. This group will be referred to as secondary care patients. The IBS Diagnosis in primary and secondary care is generally made in accordance with the Rome- III criteria, in the absence of red flag risk factors. The third and fourth groups consisted of frequency matched on age- and gender-matched control group without IBS, twice the size of the first and second group, who were randomly drawn from the Achmea database: the third group matched the primary care patients, the fourth group matched the secondary care patients. Control-group subjects all had one or more medical diagnoses registered by either GP or specialist, for any medical condition but IBS. Patients from the JPCN database were linked to an Achmea policy holder based on their date of birth, gender and postal code.

### Study period

For secondary care, we allowed the diagnosis of IBS to be made anywhere between 2006 and 2009. To ensure that a primary care patient was not subsequently referred to a hospital, we excluded all the primary care patients who were diagnosed during the last year of study. For the control subjects, the date of diagnosis of their matched case was used as an index date.

### Outcome

The primary outcomes were the direct medical costs for diagnosis and management of IBS, generated in primary and secondary care. The secondary outcomes were direct total medical costs for co-morbid chronic, functional and all other disorders of the IBS patients in the present study.

### Cost specifications

The number of contacts with the GP were divided both in primary and in secondary care for: consultations and home visits during office hours, consultations and home visits outside office hours, and repeat prescriptions.

Total costs for specialist care for IBS were calculated and the specified costs for the following three diagnostic groups: chronic disorders, all other functional disorders and all other disorders, were calculated both for primary and in secondary care .

For chronic disorders, the list of the ten most prevalent chronic diseases in the Netherlands was used (RIVM 2010): diabetes mellitus, arthritis, coronary diseases, noise- and old-age-related deafness, visual disturbances, asthma, contact eczema, chronic obstructive pulmonary disease (COPD), stroke, and constitutional eczema. Ten specialists were asked to select the DBC codes that were used to register these disorders. For the functional disorders, the same procedure was followed: specialists on different clinical topics were asked to determine which of the DBC codes concerned functional disorders. A functional disorder was defined according to the definition of Henningsen et al. [1]. The group of all other disorders excluded all previously specified groups, including psychiatric disorders (in the Netherlands these were not registered using the same system).

We included total medication costs and specified the costs both for primary and in secondary care for the following subgroups of medications associated with functional (gut) disorders: laxatives , spasmolytics, anti-depressants and hypnotics.

### Analysis

For IBS patients diagnosed in primary and secondary care as well as for their respective controls, the reimbursement data for IBS management and relevant co-morbidity were extracted and the direct medical costs were calculated; all observed years before and after the diagnosis between 2006 and 2009 were analysed as separate observations. Total costs were divided into three subgroups: GP costs, hospital or specialist costs and medication reimbursement costs. For the cases and their controls, we calculated the average annual costs before and after IBS diagnosis.

Data analysis was performed using SAS version 9.2 (SAS Institute Inc., Cary, NC). We described the differences between primary and secondary care IBS patients and their controls and tested the differences in mean costs between primary and secondary care IBS patients before and after the IBS diagnosis was determined using Student’s *t*-tests.

## Results

### Patients

Among the 20.000 ACHMEA insured primary care persons in the JCPN population we identified 326 patients diagnosed with IBS and only treated in primary care. From the 1.000.000 Achmea insured persons we were able to select 9274 IBS patients diagnosed in secondary care. We included 652 primary care and 18,548 secondary care non-IBS patients as controls. For the percentages of females and mean age, see Table 1. For a breakdown of the number of cases per year before and after IBS diagnosis, see Table 1.

**Table 1:** Study population, number and characteristics

		Primary care patients	Primary care controls	Secondary care patients	Secondary care controls
Total number	N	326	652	9274	18548
Female gender	N / %	230 / 70 %	460 / 70 %	6506 / 70 %	13012 / 70 %
Patients with data before diagnosis	N	133	266	5972	11944
Patients with data after diagnosis	N	326	652	7538	15076
Age	Average (SD)	49 (17)	49 (17)	53 (18)	53 (18)

Nine primary care control patients and one primary care IBS patient were excluded from the analysis because their annual average healthcare costs exceeded five standard deviations (SD) above the average.

### Total annual health care costs before and after IBS diagnosis

Mean total annual healthcare costs for primary care IBS patients increased after the diagnosis by 486 Euro (±3192) and for secondary care IBS patients by 2328 Euro (±5888). The difference in total cost increase between primary and secondary care patients was significant (*p* < 0.01). We did not find a significant difference in the total change in cost between the two control groups (*p* = 0.26) (Table 2).

**Table 2:** Mean healthcare costs per patient per year (mean (SD) in Euro) (including both total and specific costs) for primary and secondary care IBS patients and matched controls in the years before and after the diagnosis of IBS

		T = −3 Mean (SD)	T = −2 Mean (SD)	T = −1 Mean (SD)	Mean annual before (SD)	T = +1 Mean (SD)	T = +2 Mean (SD)	T = +3 Mean (SD)	Mean annual after (SD)	Mean dif (SD)	*P* value difference
GPs	Primary care patients	–	98 (103)	104 (103)	102 (103)	129 (120)	125 (130)	127 (127)	127 (125)	25 (121)	0.15
	Secondary care patients	93 (90)	102 (97)	142 (119)	122 (110)	147 (165)	155 (158)	168 (181)	154 (166)	32 (146)	
	Primary care controls	–	67 (67)	74 (94)	72 (87)	68 (78)	68 (75)	75 (95)	70 (81)	−2 (82)	0.13
	Secondary care controls	74 (88)	77 (99)	78 (98)	77 (97)	78 (96)	80 (95)	83 (96)	80 (2)	3 (96)	
Hospital specialists^a^	Primary care patients	–	1168 (2645)	1087 (2520)	1111 (2551)	1345 (2572)	1391 (2932)	1544 (2822)	1409 (2758)	298 (2719)	<0.01^×^
	Secondary care patients	1139 (3780)	1063 (2763)	1500 (3713)	1303 (3450)	3695 (6400)	2678 (6198)	2815 (5783)	3173 (6230)	1870 (5269)	
	Primary care controls	–	591 (1812)	874 (2504)	792 (2325)	822 (2328)	1048 (2882)	1051 (2553)	955 (2585)	164 (2537)	0.16
	Secondary care controls	1259 (4742)	1319 ± 4806)	1342 (6006)	1322 (5457)	1294 (8598)	1333 (4987)	1441 (5071)	1337 (5149)	15 (5277)	
Medication	Primary care patients	–	401 (657)	448 (680)	434 (672)	522 (792)	580 (911)	751 (2087)	598 (1269)	164 (1177)	<0.01^×^
	Secondary care patients	477 (992)	545 (1143)	630 (1227)	579 (1169)	939 (1707)	999 (1797)	1163 (2087)	1005 (1822)	426 (1587)	
	Primary care controls	–	398 (783)	481 (1360)	457 (1220)	449 (1252)	471 (1231)	490 (1435	466 (1291)	10 (1278)	0.97
	Secondary care controls	525 (1398)	555 (1390)	558 (1452)	552 (1434)	561 (1597)	558 (1346)	561 (1304)	560 (1459)	8 (1445)	
Total^a^	Primary care patients	–	1667 (2922)	1640 (2898)	1648 (2898)	1996 (2923)	2096 (3269)	2423 (3746)	2134 (3258)	486 (3192)	<0.01^×^
	Secondary care patients	1708 (4080)	1710 (3204)	2272 (4145)	2003 (3863)	4781 (7058)	3832 (6951)	4146 (6670)	4331 (6958)	2328 (5888)	
	Primary care controls	–	1056 (2236)	1429 (3052)	1320 (2840)	1338 (2872)	1586 (3369)	1616 (3156)	1492 (3120)	171 (3068)	0.26
	Secondary care controls	1858 (5295)	1950 (5376)	1978 (6501)	1951 (6002)	1933 (5893)	1971 (5490)	2086 (5558)	1976 (5692)	26 (5812)	

^a^Excluding psychiatric care; ×, significant difference at alpha = 0.05

T = −3 indicates three years before diagnosis, T = +1 one year after diagnosis and so forth

This increase was primarily explained by the increase in hospital specialists’ costs and in medication costs. These costs increased and remained high over each of the three years after diagnosis. There was a slight increase in costs for GP care for primary and secondary care patients, whereas GP costs in the two control groups minimally changed over time. The increase in costs was significantly greater for secondary care patients compared to primary care patients for medications, hospital specialist costs and total costs, while there was no significant difference for GP costs (Table 2).

### Number of contacts with the general practitioner before and after IBS diagnosis

The mean annual number of consultations and home visits during office hours increased by 2 visits for primary care and 1 visits for secondary care IBS patients after the IBS diagnosis (Table 3). For control patients these numbers did not change. Consultations outside of office hours and home visits increased only for secondary care IBS patients, whereas for controls there were no consultations.

**Table 3:** Annual number of GP contacts (mean (SD)) for primary and secondary care IBS patients and matched controls specified per year before and after the diagnosis of IBS

		T = −3 Mean (SD)	T = −2 Mean (SD)	T = −1 Mean (SD)	Mean annual before (SD)	T = +1 Mean (SD)	T = +2 Mean (SD)	T = +3 Mean (SD)	Mean annual after Mean (SD)	Mean dif Mean (SD)
1. Consults and home visits during office hours	Primary care patients	–	6 (6)	7 (6)	6 (6)	8 (6)	8 (7)	8 (7)	8 (6)	2 (6)
	Secondary care patients	6 (6)	7 (6)	10 (7)	8 (6)	9 (8)	9 (8)	10 (9)	9 (8)	1 (7)
	Primary care controls	–	4 (4)	5 (5)	4 (5)	4 (5)	4 (5)	5 (6)	4 (5)	0 (5)
	Secondary care controls	5 (5)	5 (6)	5 (5)	5 (5)	5 (5)	5 (5)	5 (6)	5 (5)	0 (5)
2. Consults and home visits outside office hours	Primary care patients	–	0 (1)	0 (1)	0 (1)	0 (1)	0 (1)	0 (0)	0 (1)	0 (1)
	Secondary care patients	0 (1)	0 (1)	0 (1)	0 (1)	1 (2)	1 (1)	1 (2)	1 (2)	0 (1)
	Primary care controls	–	0 (0)	0 (1)	0 (1)	0 (0)	0 (1)	0 (1)	0 (1)	0 (1)
	Secondary care controls	0 (1)	0 (1)	0 (1)	0 (1)	0 (1)	0 (1)	0 (1)	0 (1)	0 (1)
3. Repeat prescriptions	Primary care patients	–	4 (6)	4 (5)	4 (6)	5 (7)	5 (7)	6 (8)	5 (7)	2 (7)
	Secondary care patients	4 (6)	5 (6)	5 (6)	5 (6)	6 (7)	7 (8)	7 (8)	7 (7)	2 (7)
	Primary care controls	–	4 (6)	4 (6)	4 (6)	4 (5)	4 (5)	4 (6)	4 (5)	0 (5)
	Secondary care controls	4 (5)	4 (6)	4 (6)	4 (6)	4 (6)	4 (6)	4 (6)	4 (6)	0 (6)

All p-values for the difference in means were not significant (p > 0.5). T = −3 indicates three years before diagnosis, T = +1 one year after diagnosis and so forth

For primary and secondary care IBS patients the mean annual number of repeat prescriptions increased by 2, whereas for controls this remained at the same level (Table 3).

### Annual hospital specialist care costs before and after IBS diagnosis

Mean overall costs for specialist care for secondary care IBS patients rose by 1870 Euro (±5269) annually after the IBS diagnosis was made. For primary care IBS patients this increase was 298 Euro (±2719). This difference between primary and secondary care patients was significant (*p* < 0.01) (Table 2).

For secondary care IBS patients, the mean IBS specific, annual specialists’ care costs increased with 427 Euro (±572).

Mean annual costs for all other chronic disorders increased with 100 Euro (±1299) for primary care IBS patients and with 282 Euro (±2337) for secondary care patients The cost increase was significantly higher in secondary care (*p* <0.01)

The difference in the increase of costs between primary and secondary care patients was significant (*p* < 0.01) (Table 4). These costs remained high over each of the three years after diagnosis. The disease groups contributing to the cost increase were: angina pectoris, arthritis and stroke in primary care patients and coronary diseases, COPD, asthma and visual disturbances in secondary care patients.

**Table 4:** Mean health care costs per patient per year for primary and secondary care IBS patients and their matched controls specified per year before and after diagnosis for IBS, chronic disorders, functional disorders and all other disorders (mean (SD) in Euro)

		T = −3 Mean (SD)	T = −2 Mean (SD)	T = −1 Mean (SD)	Mean annual before Mean (SD)	T = +1 Mean (SD)	T = +2 Mean (SD)	T = +3 Mean (SD)	Mean annual after Mean (SD)	Mean dif Mean (SD)	*p* value difference^∂^
Irritable bowel syndrome	Primary care patients	–	0 (0)	0 (0)	0 (0)	0 (0)	0 (0)	0 (0)	0 (0)	0 (0)	<0.01^×^
	Secondary care patients	0 (0)	0 (0)	0 (0)	0 (0)	725 (866)	174 (520)	175 (468)	427 (745)	427 (572)	
	Primary care controls	–	0 (0)	0 (0)	0 (0)	0 (0)	0 (0)	0 (0)	0 (0)	0 (0)	0.99
	Secondary care controls	0 (0)	0 (0)	0 (0)	0 (0)	0 (0)	0 (0)	0 (0)	0 (0)	0 (0)	
Chronic disorders	Primary care patients	–	332 (2074)	161 (543)	211 (1213)	289 (1151)	271 (1063)	407 (1819)	312 (1319)	100 (1299)	<0.01^×^
	Secondary care patients	318 (2373)	326 (1649)	379 (2017)	352 (1967)	600 (2659)	652 (2522)	684 (2407)	635 (2564)	282 (2337)	
	Primary care controls	–	189 (1095)	236 (1295)	222 (1239)	212 (1137)	309 (1600)	413 (1924)	294 (1523)	72 (1473)	0.38
	Secondary care controls	385 (2120)	398 (2314)	354 (1783)	373 (2021)	395 (2926)	379 (2187)	411 (2500)	393 (2611)	20 (2386)	
Functional disorders	Primary care patients	–	21 (83)	67 (390)	54 (330)	30 (236)	41 (228)	17 (141)	31 (214)	−23 (241)	<0.01^×^
	Secondary care patients	27 (242)	32 (262)	40 (289)	36 (274)	55 (346)	58 (478)	60 (365)	57 (399)	21 (353)	
	Primary care controls	–	42 (440)	6 (57)	17 (242)	12 (134)	9 (96)	13 (112)	11 (117)	−6 (149)	0.32
	Secondary care controls	17 (214)	18 (218)	17 (182)	17 (199)	19 (223)	16 (151)	15 (136)	17 (185)	0 (191)	
All other disorders^a^	Primary care patients	–	815 (1752)	860 (2382)	847 (2210)	1026 (2312)	1080 (2585)	1120 (2215)	1067 (2384)	221 (2351)	<0.01^×^
	Secondary care patients	794 (2863)	705 (2064)	1081 (2828)	915 (2616)	2315 (5429)	1794 (5322)	1896 (4882)	2054 (5292)	1139 (4395)	
	Primary care controls	–	360 (954)	633 (2005)	553 (1767)	598 (1880)	730 (2234)	625 (1407)	650 (1911)	97 (1885)	0.26
	Secondary care controls	857 (4107)	903 (3968)	971 (5630)	932 (4925)	880 (4197)	937 (4294)	1016 (4212)	927 (4233)	5 (4530)	

T = −3 indicates three years before diagnosis, T = +1 one year after diagnosis and so forth

^a^Excluding irritable bowel syndrome, chronic disorders, functional disorders and psychiatric disorders; ×: significant difference at alpha = 0.05; ∂: *p* value for the difference in increase of costs between primary and secondary care subjects

In both IBS patient groups, no substantial change in costs related to all other functional disorders after the diagnosis (-23 and +21 respectively) was found , though this difference in the increase of costs between primary and secondary care patients was significant (*p* < 0.01).

There was a considerable increase in the mean annual costs for ‘all other disorders’ after IBS was diagnosed in primary care patients (221 Euro (±2351)). These costs also increased and remained high over each of the three years after diagnosis. Highest increases were observed in costs for hernia cicatricles, endometriosis and ileus. For secondary care patients, the costs increased by 1139 Euro (±4395). The largest increases were observed for diverticulosis, colon cancer, breast cancer and urinary bladder tumours. This difference between primary and secondary care patients was significant (*p* < 0.01) (Table 4).

### Medications before and after IBS diagnosis

The total costs for medications increased significantly by 426 Euro (±1587) in the group of secondary care patients and 164 Euro (±1177) for primary care patients (Table 2). This difference between primary and secondary care patients was significant (*p* < 0.01). Table 5 shows the change in medication costs in medication subgroups. There was a sharp increase in antacid use among the secondary care patients. The main contribution to the medication costs increase came from ‘All other drugs’, not related to any specific type of gastrointestinal medication. In this group, the difference in the cost increase between primary and secondary care patients was significant (*p* = 0.02).

**Table 5:** Mean costs per patient per year for medication subgroups (mean euro (SD)) of primary and secondary care IBS patients and their matched controls specified per year before and after diagnosis

		T = −3 Mean (SD)	T = −2 Mean (SD)	T = −1 Mean (SD)	Mean annual before Mean (SD)	T = +1 Mean (SD)	T = +2 Mean (SD)	T = +3 Mean (SD)	Mean annual after Mean (SD)	Mean dif Mean (SD)	*p* value difference
1. Laxatives	Primary care patients	–	10 (38)	13 (41)	12 (40)	32 (105)	34 (107)	39 (123)	34 (110	23 (101)	0.72
	Secondary care patients	9 (48)	9 (40)	18 (45)	14 (44)	46 (76)	33 (76)	36 (83)	39 (78)	25 (66)	
	Primary care controls	–	1 (7)	3 (14)	2 (12)	3 (17)	2 (13)	4 (22)	3 (17)	1 (17)	
	Secondary care controls	5 (28)	5 (28)	5 (29)	5 (28)	5 (30)	6 (31)	6 (32)	6 (31)	0 (30)	
2. Spasmolytics	Primary care patients	–	6 (24)	6 (24)	6 (24)	11 (31)	7 (25)	9 (33)	10 (29)	4 (28)	0.25
	Secondary care patients	12 (70)	13 (75)	14 (76)	13 (75)	14 (73)	16 (77)	16 (76)	15 (75)	2 (75)	
	Primary care controls	–	0 (0)	0 (2)	0 (2)	0 (6)	0 (3)	0 (3)	0 (4)	0 (4)	0.99
	Secondary care controls	6 (52)	7 (53)	7 (52)	7 (52)	7 (52)	8 (53)	7 (47)	7 (51)	0 (52)	
3. Anti-depressants	Primary care patients	–	7 (45)	10 (45)	9 (45)	15 (84)	17 (100)	20 (100)	16 (94)	7 (86)	0.66
	Secondary care patients	2 (13)	2 (16)	5 (20)	3 (18)	9 (29)	8 (29)	9 (32)	8 (30)	5 (25)	
	Primary care controls	–	2 (11)	4 (38)	4 (33)	8 (49)	10 (67)	8 (51)	9 (56)	5 (53)	0.02^×^
	Secondary care controls	1 (9)	1 (9)	1 (10)	1 (10)	1 (9)	1 (9)	1 (11)	1 (10)	0 (10)	
4. Antacids	Primary care patients	–	83 (229)	72 (181)	75 (196)	76 (184)	70 (155)	60 (138)	70 (164)	−5 (171)	<0.01^×^
	Secondary care patients	47 (132)	56 (139)	70 (153)	62 (146)	117 (214)	113 (204)	115 (204)	115 (209)	54 (186)	
	Primary care controls	–	26 (98)	22 (78)	23 (84)	27 (115)	30 (112)	29 (106)	28 (112)	5 (107)	0.16
	Secondary care controls	36 (120)	37 (115)	36 (114)	36 (115)	36 (116)	35 (110)	33 (106)	35 (112)	−1 (113)	
5. Hypnotics	Primary care patients	–	12 (38)	8 (29)	10 (32)	8 (32)	7 (29)	4 (19)	7 (28)	−3 (29)	<0.01^×^
	Secondary care patients	8 (30)	9 (33)	9 (34)	9 (33)	12 (41)	13 (42)	11 (40)	12 (41)	3 (38)	
	Primary care controls	–	6 (23)	5 (22)	5 (22)	6 (27)	5 (25)	3 (19)	5 (25)	0 (24)	0.99
	Controls to referred	6 (29)	6 ± 27)	6 (27)	6 (27)	6 (28)	6 (28)	5 (26)	5 (28)	0 (28)	
6. Other ATC group N drugs^a^	Primary care patients	–	55 (139)	45 (152)	48 (148)	53 (144)	71 (234)	75 (274)	64 (214)	16 (203)	0.40
	Secondary care patients	83 (309)	82 (305)	90 (357)	86 (334)	106 (397)	115 (410)	120 (412)	112 (404)	26 (377)	
	Primary care controls	–	28 (79)	56 (311)	47 (265)	64 (456)	65 (359)	65 (352)	65 (400)	17 (378)	0.29
	Secondary care controls	55 (246)	60 (292)	60 (319)	59 (300)	64 (354)	58 (306)	58 (293)	61 (327)	1 (316)	
7. Other ATC group A drugs^b^	Primary care patients	–	44 (183)	42 (194)	43 (191)	40 (178)	41 (134)	46 (141)	42 (155)	−1 (163)	<0.01^×^
	Secondary care patients	20 (97)	22 (95)	28 (125)	25 (112)	64 (223)	75 (257)	99 (304)	75 (253)	50 (207)	
	Primary care controls	–	29 (97)	35 (146)	33 (133)	32 (170)	36 (195)	24 (120)	32 (169)	−2 (163)	0.88
	Secondary care controls	42 (182)	45 (193)	45 (186)	45 (188)	43 (182)	45 (188)	42 (185)	44 (185)	−1 (186)	
8. All other drugs^c^	Primary care patients	–	182 (321)	252 (454)	232 (419)	287 (508)	333 (649)	497 (1944)	354 (1085)	123 (992)	0.02^×^
	Secondary care patients	296 (830)	352 (1009)	397 (1065)	367 (1015)	570 (1504)	627 (1565)	757 (1859)	627 (1604)	261 (1392)	
	Primary care controls	–	306 (678)	356 (1263)	341 (1124)	308 (1082)	322 (1070)	356 (1320)	325 (1140)	−17 (1137)	0.57
	Secondary care controls	373 (1258)	393 (1235)	397 (1295)	392 (1270)	399 (1464)	401 (1202)	410 (1152)	402 (1320)	9 (1299)	

T = −3 indicates three years before diagnosis, T = +1 one year after diagnosis and so forth

×Significant difference at alpha = 0.05

ATC group N drugs

^a^ATC group N drugs: Drugs typically aimed at the neurological system, excluding Anti-depressants and Hypnotics

^b^ATC group A drugs: Drugs typically aimed at the alimentary tract, excluding laxatives, spasmolytics and antacids

^c^Excluding ATC group A and group N drugs

## Discussion

### Summary of findings

Total health care costs for IBS patients increased substantially in the years after the diagnosis, 29 % for primary and 116 % for secondary care patients. In secondary care this increase was primarily attributed to costs for hospital specialists (+144 %) and medications (+74 %). The most remarkable is that for all three kinds of medical costs these remain high all three years after diagnosis.

The additional specialist-associated costs for the primary care IBS patients were primarily due to chronic disorders other than IBS (nearly 50 %) and by other disorders (25 %). This observation is consistent with the results from a study by Levy et al. [11], in which the majority of the excess health care costs between IBS patients and population controls was attributed to care unrelated to lower GI problems. The increase in costs related to hospital specialist care for secondary care patients is, for the most part, due to costs for other chronic disorders (+80 %) and ‘all other disorders’ (+124 %). Patients with functional gastrointestinal diseases consult their doctors more often for non-gastrointestinal complaints [12] and for other somatic and psychiatric disorders [3]. We hypothesise that gastroenterologists are more likely to refer IBS patients to other hospital specialists than GPs, thus explaining the differences in increased costs. In the Netherlands, as in many other countries with a strong primary care, the GP as acts as the ‘case manager ‘of the patient , coordinating the diagnostic and treatment process. The longitudinal relation with the patient and the knowledge of medical history and psychosocial system facilitates an integral approach to IBS , and helps to prevent unnecessary referral and diagnostic procedures. This benefits both disease outcome, and patient’s quality of life and reduces health care costs.

### Comparison with similar studies

It is remarkable that in our study, the costs for other functional disorders (e.g., fibromyalgia, fatigue and unexplained pain) in IBS patients are not very high, although high rates of co-occurrence of IBS with other functional diseases have been reported [3]. In the Netherlands, both in primary and secondary care, only one diagnosis per specialty can be designated per treatment. The general practitioner and specialist may be inclined to select the diagnosis with the highest reimbursement value as a result of this policy. Because the reimbursement value for functional disorders is relatively low, this result might lead to an under diagnosis.

The increase in antacid use after the IBS diagnosis in secondary care may be explained by the fact that more than in primary care , gastroenterologists are aware of the overlap between IBS and upper gastrointestinal symptoms. This results is frequent co-prescription of antacids, H 2 blockers and PPI’s in patients diagnosed with IBS.

### Strengths and limitations

The extrapolated incidence of IBS in our study population was 5. 4/1000/year, which is in line with the IBS incidence reported for primary care in the Netherlands. This indicates that the patients sample we extracted is representative for primary care IBS patient group in the Netherlands.

A strong point of this study is the long observation period. Whereas the majority of previous studies have gathered information for up to one year [7, 13–17], we gathered total costs over four years. Because data from the insurance company are based on reimbursement, the dataset provide insight into valid medical costs and not proxy costs calculated from medical file research or questionnaire data.

The demographic characteristics of our IBS population are notable. The Achmea population is slightly older than the mean Dutch population. In the Netherlands, approximately 15 % of the population is 65 years or older; for the Achmea population, this is 18 % [18]. In the studies of Akehurst et al. [7], Creed et al. [13] and Hillillä et al. [16], the mean ages of the studied populations were 47, 39 and 42 years, respectively. The mean age of our IBS population (i.e. 49 for primary care; 53 for secondary care) is higher. Total health care costs increase with age and decrease with a higher educational level [19]. College-educated subjects incur significantly lower health care costs through their insurance program [19]. The Achmea population has a lower educational level (as measured by the postal code) compared to the average Dutch population.

Costs observed in the present study were highly skewed towards the lower end of the cost spectrum, as reflected in the high standard deviations relative to the average costs. One might argue that the median values would be more suitable for presenting numbers in highly skewed distributions. However, we chose not to do so because in healthcare, a small group of people are often responsible for a large share of the costs. The medians might result in values that are very low or even zero, underestimating the actual financial burden of IBS on society.

Not all patients in our study had data available for the year before the IBS diagnosis. Nevertheless, we calculated averages for these patients for the period after the initial diagnosis because the number of primary care patients with IBS was low. One could argue that this observation may have generated a bias because, theoretically, patients for whom data are only available after diagnosis have different characteristics than those who were analysed both in the years before and after diagnosis. To test this possibility, we examined only the cases for whom data were available before and after the diagnosis, and we did not arrive at different conclusions.

Another limitation is the fact that although multidisciplinary guidelines in the Netherlands recommend the use of Rome criteria to diagnose IBS, we did not check if the physicians in our study actually used these criteria. Although this may have resulted in diagnostic uncertainty in some, we think that the diagnosis was valid in the majority of IBS patients included.

One might argue that there is a difference in IBS symptom severity and disease impact between patients attending primary and secondary care, which explains the bigger increase in IBS costs in secondary care. We do not share this viewpoint. A study by Smith et al. showed [20] no difference in IBS symptom severity between patients treated in primary and secondary care. In addition, our results demonstrate that the difference in costs between primary and secondary care is not so much due to direct IBS related costs but due to costs for other disorders.

## Conclusion

Health care costs substantially increase after the diagnosis of IBS is made, and costs increase significantly more and remain higher over the years for patients who are treated in secondary care compared to patients treated by a GP. IBS patients should be treated in primary-care where possible, not only because guidelines recommend this from a quality of care viewpoint, but also to optimize use of health care resources. Referral should be restricted to those patients with alarm symptoms, with ill-matching symptoms, or other cases of diagnostic uncertainty.

## Ethics

The use of study data was approved by the research committees of both Achmea and the Julius Primary Care Network.

## Abbreviations

ATC: Anatomical Therapeutic Codes
COPD: Chronic Obstructive Pulmonary Disease
DBC: Diagnostic Treatment Codes
EMR: Electronic Medical Record
FSS: Functional Somatic Syndrome
GERD: Gastroesophageal Reflux Disease
GP: General Practitioner
IBS: Irritable Bowel Syndrome
ICPC- codes: International Classification for Primary Care
JPCN: Julius Primary Care Network
RIVM: Rijksinstituut voor Volksgezondheid en Milieu
UK: United Kingdom
USA: United States of America
USD: United States dollar
PPI: Proton Pump Inhibitor
